# Transplant Trial Watch

**DOI:** 10.3389/ti.2024.12711

**Published:** 2024-02-08

**Authors:** Simon R. Knight, John M. O’Callaghan

**Affiliations:** ^1^ Oxford Transplant Centre, Churchill Hospital, Oxford, United Kingdom; ^2^ Centre for Evidence in Transplantation, Nuffield Department of Surgical Sciences, University of Oxford, Oxford, United Kingdom; ^3^ University Hospitals Coventry and Warwickshire, Coventry, United Kingdom

**Keywords:** kidney transplantation, hypothermic machine perfusion, randomised controlled trial, lung transplantation, belatacept

To keep the transplantation community informed about recently published level 1 evidence in organ transplantation ESOT and the Centre for Evidence in Transplantation have developed the Transplant Trial Watch. The Transplant Trial Watch is a monthly overview of 10 new randomised controlled trials (RCTs) and systematic reviews. This page of Transplant International offers commentaries on methodological issues and clinical implications on two articles of particular interest from the CET Transplant Trial Watch monthly selection. For all high quality evidence in solid organ transplantation, visit the Transplant Library: www.transplantlibrary.com.

RANDOMISED CONTROLLED TRIAL 1 Perfusate Proteomes Provide Biological Insight Into Oxygenated Versus Standard Hypothermic Machine Perfusion in Kidney Transplantation.

*by Mulvey, J. F., et al. Annals of Surgery 2023; 278(5): 676–682.*

## Aims

The aim of this study was to provide mechanistic insight into biological alterations that occur in deceased donor kidneys during standard non-oxygenated versus oxygenated hypothermic machine perfusion (HMP), using perfusate samples collected in the COMPARE study.

## Interventions

In the COMPARE trial, pairs of kidneys donated following circulatory death were randomly assigned to receive either oxygenated HMP or non-oxygenated HMP.

## Participants

210 perfusate samples.

## Outcomes

The main outcome of this paper was to identify protein changes across durations of perfusion and in relation to 12-month estimated glomerular filtration rate (eGFR).

## Follow-Up

12 months.

## CET Conclusion

*by John O’Callaghan*

This well-written report details an analysis of perfusate samples collected during the COMPARE study, an RCT comparing oxygenated with non-oxygenated machine perfusion. Mass spectrometry was used to analyse the proteomic make up of the perfusate fluid. During hypothermic machine perfusion, proteins enter the perfusate system, increasing over time. The authors explored the relation between perfusate proteins and clinical outcomes, with some indication that outcomes such as acute rejection and kidney function at 12 months.

## Trial Registration

ISRCTN32967929.

## Funding Source

Non-industry funded.

RANDOMISED CONTROLLED TRIAL 2 A Pilot Randomized Controlled Trial of *De Novo* Belatacept-Based Immunosuppression After Lung Transplantation.

*by Huang, H. J., et al. Transplantation 2023 [record in progress].*

## Aims

This study aimed to evaluate the feasibility and inform the design of an RCT investigating the efficacy and safety of belatacept following lung transplantation.

## Interventions

Participants were randomly assigned to either continue standard-of-care immunosuppression or switch to belatacept.

## Participants

27 lung transplant recipients.

## Outcomes

The primary outcome was to assess the feasibility of randomising 80% of eligible patients within 4 h posttransplantation. The primary outcome was later changed to survival following the cessation of treatment with belatacept.

## Follow-Up

1 year posttransplantation.

## CET Conclusion

*by Simon Knight*

This pilot study recruited lung transplant recipients at 2 sites, and randomised them to standard immunosuppression (Tac, MMF, Pred) or a belatacept-based regimen (Tac, Belatacept and pred). The hypothesis was that belatacept-based immunosuppression might reduce the incidence of donor-specific antibodies (DSA), leading to a reduction in the risk of chronic lung allograft dysfunction (CLAD). The study was stopped after recruitment of 27 patients due to 3 deaths in the belatacept arm. Causes of death varied—2 patients died from COVID-19 infection, one from CLAD related to infection, one from PTLD, one from pulmonary embolus and one from haemothorax. The authors ascribe 4 of these deaths to viral infections. No differences were seen in incidence of CLAD or development of DSA. It is very difficult to interpret these results given the small numbers, but clearly the authors were correct in stopping the study and switching patients to standard immunosuppression. The relationship of four of the deaths to viral infection would suggest that the immunosuppressive regimen may have contributed, and in the absence of any detectable clinical benefit, the conclusion that this regimen is unsafe in lung transplant recipients seem justified.

## Jadad Score

2.

## Data Analysis

Strict intention-to-treat analysis.

## Allocation Concealment

No.

## Trial Registration

ClinicalTrials.gov—NCT03388008.

## Funding Source

Non-industry funded.

## Clinical Impact Summary

*by Simon Knight*

Chronic Lung Allograft Dysfunction (CLAD) is an important long-to medium-term cause of morbidity and mortality following lung transplantation [1]. It results predominantly from chronic immune damage, and is associated with the formation of donor-specific antibodies (DSA) [2]. Management of CLAD is challenging once established, so most focus is on adequate immunosuppression and prevention of infection to reduce the risk of occurrence [1].

Early studies of belatacept, a T-cell co-stimulation blocker, demonstrated a significantly lower incidence of DSA-formation over 7-year post-transplant compared to a calcineurin-inhibitor-based regimen in kidney transplant recipients [3]. This led the teams in Houston and St. Louis to design a phase 2 pilot study to investigate the impact of belatacept-based immunosuppression on risk of DSA formation and CLAD in lung transplant recipients, reported in Transplantation recently [4].

The study recruited *de novo* lung transplant recipients, and randomised them to standard immunosuppression (ATG, tacrolimus, mycophenolate and prednisone) or to belatacept-based immunosuppression (tacrolimus, belatacept and prednisone). The study was stopped after recruitment of 27 of patients due to excess mortality in the belatacept arm. Overall, five of 13 patients receiving belatacept died, with one additional death after the end of follow-up. At first glance, causes of death appear varied, with two patients dying of COVID-19, one with CLAD, one post-transplant lymphoproliferative disorder (PTLD), one haemothorax and one pulmonary embolus. However, the authors note that four of six deaths had a viral association (viral CLAD, PTLD and COVID-19), with the suggestion that belatacept in this patient population may be associated with increased susceptibility to viral infection and infective complications.

It is hard to draw firm conclusions from a small number of patients, but in the absence of any noticeable difference in DSA formation or development of CLAD, this sobering experience would seem to suggest that the risk of *de novo* belatacept in lung transplant recipients far outweighs any potential theoretical benefit. Other studies have suggested that conversion to belatacept post-transplant might be feasible, but potentially with a higher risk of rejection [5, 6]. Numbers are small and more evidence is needed before belatacept-based strategies for lung recipients can be recommended.

### Clinical Impact

4/5.
